# RETRACTION: Sex‐Dependent Paracrine Effect of Conditioned Media From Adipose Tissue Derived Mesenchymal Stem Cells on Prostate Cancer Cells

**DOI:** 10.1111/jcmm.71001

**Published:** 2025-12-22

## Abstract

**RETRACTION**: MirzaeiA. , MashhadiR. , AghsaeifardZ. , IzadiM. , DougahehS. N. H. , OmidR. , GuitynavardF. , NikoofarP. , and AghamirS. M. K. , “Sex‐Dependent Paracrine Effect of Conditioned Media From Adipose Tissue Derived Mesenchymal Stem Cells on Prostate Cancer Cells,” Journal of Cellular and Molecular Medicine 29, no. 9 (2025): e70569, 10.1111/jcmm.70569.40356028 PMC12069013

The above article, published online on 12 May 2025 in Wiley Online Library (wileyonlinelibrary.com), has been retracted by agreement between; the authors; the journal Editor‐in‐Chief, Stefan N. Constantinescu; the Foundation for Cellular and Molecular Medicine; and John Wiley & Sons Ltd. The retraction has been agreed upon following concerns raised by a third‐party regarding image duplication. The investigation confirmed an overlap between the LNCaP MCM and FCM images in Figure 2. In addition, the DU145 and LNCaP control images in Figure 3 were found to be identical. The authors fully cooperated with the investigation, explaining that the duplication was due to an inadvertent error in figure preparation. They also provided some data; however, the data provided was insufficient to restore the editor's confidence in the results and conclusions. Consequently, the editors consider the findings unreliable.

**RETRACTION**: A. Mirzaei , R. Mashhadi , Z. Aghsaeifard , M. Izadi , S. N. H. Dougaheh , R. Omid , F. Guitynavard , P. Nikoofar , and S. M. K. Aghamir , “Sex‐Dependent Paracrine Effect of Conditioned Media From Adipose Tissue Derived Mesenchymal Stem Cells on Prostate Cancer Cells,” Journal of Cellular and Molecular Medicine 29, no. 9 (2025): e70569, 10.1111/jcmm.70569.40356028 PMC12069013

The above article, published online on 12 May 2025 in Wiley Online Library (wileyonlinelibrary.com), has been retracted by agreement between; the authors; the journal Editor‐in‐Chief, Stefan N. Constantinescu; the Foundation for Cellular and Molecular Medicine; and John Wiley & Sons Ltd. The retraction has been agreed upon following concerns raised by a third‐party regarding image duplication. The investigation confirmed an overlap between the LNCaP MCM and FCM images in Figure 2. In addition, the DU145 and LNCaP control images in Figure 3 were found to be identical. The authors fully cooperated with the investigation, explaining that the duplication was due to an inadvertent error in figure preparation. They also provided some data; however, the data provided was insufficient to restore the editor's confidence in the results and conclusions. Consequently, the editors consider the findings unreliable.

